# Supplementary material for: van der Waals driven anharmonic melting of the 3D charge density wave in VSe2
Source: Nat Commun. 2021 Jan 26;12:598. doi: 10.1038/s41467-020-20829-2 (PMC7838422; doi:10.1038/s41467-020-20829-2)
Supplement: Supplementary file 1 — Supplementary Information [file 41467_2020_20829_MOESM1_ESM.pdf]

Supplementary Information for

van der Waals driven anharmonic melting of the 3D CDW in VSe_2

Josu Diego, A.H. Said, S.K. Mahatha, Raffaello Bianco, Lorenzo Monacelli, Matteo Calandra, Francesco Mauri, K. Rossnagel, Ion Errea* and S. Blanco-Canosa*

**ion.errea@ehu.eus*
** sblanco@dipc.org*

Materials and Methods.

1) Sample characterization

High quality single crystals of VSe_2 were synthesized by chemical vapor transport using iodine as transport agent. Bulk VSe_2 crystallizes in a 1T polytype , with the V atoms covalently bonded with a distorted octahedra of Se atoms to form layers of VSe_2, stacked along the (0001) direction mediated by van der Waals interactions, (Supplementary Figure S1(a)). The chemical composition and cystallinity of our single crystals are confirmed by energy dispersive x-ray analysis (EDX) , which shows a 1:2 atomic ratio of V:Se (Supplementary Figure S1(b)) and the observation of out-of- plane Bragg peaks, (Supplementary Figure S1(c)). The lattice parameters: $a=b= 3.46$ Å, $c= 6.09$ Å; $\alpha=\beta=90º$, $\gamma=120º$ were refined from the alignment of the 004 and 202 Bragg peaks of the VSe_2 crystal in the IXS spectrometer and, therefore, used to define the orientation matrix.

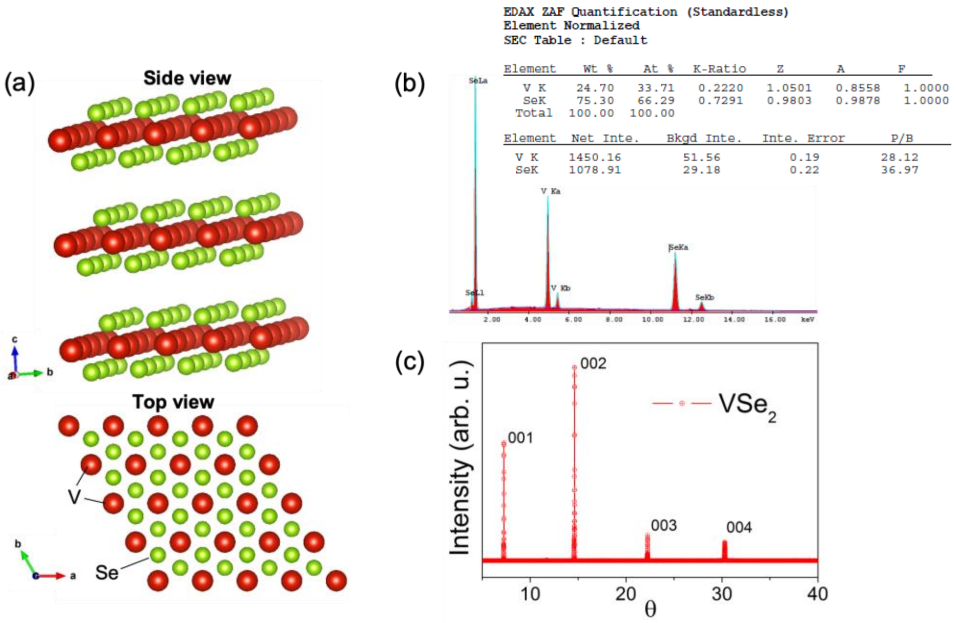

Supplementary Fig. 1: **Structural characterization of VSe_2.** (a) Layered structure of VSe_2, trigonal $P\bar{3}m1$, space group nº 164. (b) Energy dispersive x-ray (EDX) analysis, showing the 1:2 atomic ratio of VSe_2. (c) Specular diffraction pattern of VSe_2 flake, out of plane oriented, displaying the Bragg peaks along the *c*-direction.

The accurate determination of the charge density wave onset was obtained by thermodynamic measurements. In Supplementary Figure S2, we show both the magnetization and transport measurements of VSe_2. Magnetic measurements were performed in a PPMS-VSM magnetometer. The temperature dependence of the resistance (*R*) shows an onset of the CDW at 110 K, as evidenced by the hump in *R*(*T*).

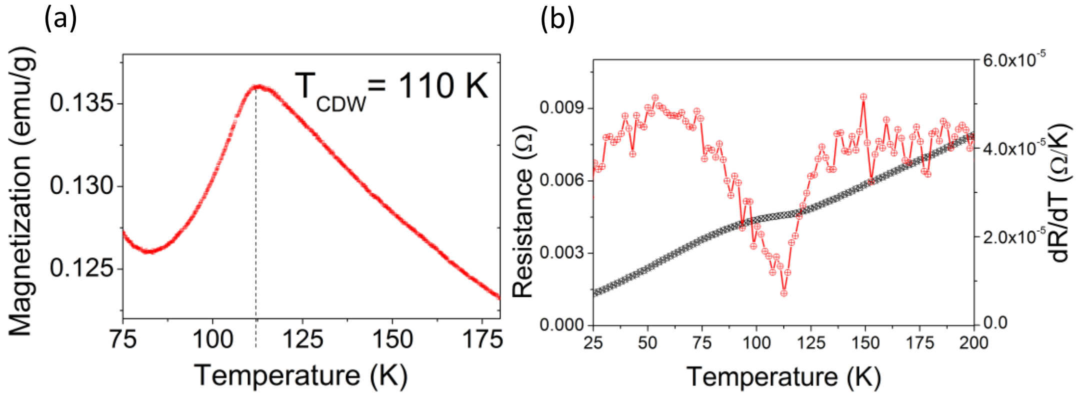

Supplementary Fig. 2: **Magnetic and electric characterization of VSe_2**. (a) Magnetization and (b) Resitance vs Temperature of VSe_2. The CDW transition is set at 110 K.

2) Inelastic x-ray scattering (IXS)

High resolution inelastic x-ray scattering measurements were carried out at the HERIX spectrometer at the 30-ID beamline of the Advanced Photon Source (APS), Argonne National Laboratory. The incident beam energy was 23.72 keV, and the horizontally scattered beam was analyzed by a dice spherical silicon analyzer, Si (12, 12, 12). The energy beam and momentum resolution was 1.5 meV and 0.65 nm^{-1}. Supplementary Figure S3 shows the resolution function of the spectrometer, fitted to a Pseudo-Voigt profile:

$$y = y_0 + A\left(\mu \times \frac{2}{\pi}\frac{w_L}{4x^2+w_L^2} + (1-\mu) \times \sqrt{\frac{4\ln 2}{\pi}}\frac{e^{-4\ln(2)x^2/w_G}}{w_G}\right) \quad \text{Supplementary Eq. (1)}$$

where w_L, w_G and μ are the Lorentzian and Gaussian linewidths and the Lorentz factor, respectively (w_L= 1.516, w_G= 1.528 and μ= 0.457).

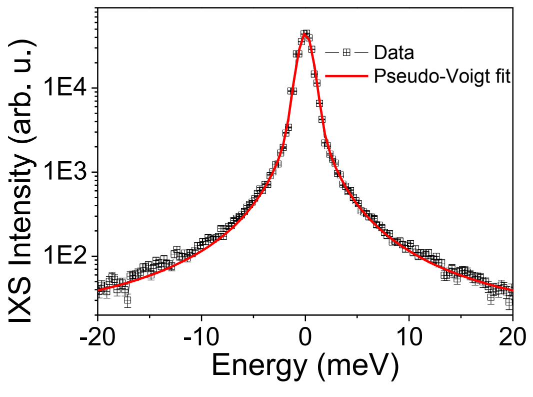

Supplementary Fig. 3: **Resolution function of IXS**. Experimentally determined resolution function of the analyzer 9 at HERIX and its fitting to a Pseudo-Voigt profile.

2.1) Quasi-elastic central peak

The IXS spectra is composed of inelastic part coming from phonons and an elastic signal at E_{loss}=0 resulting from incoherent scattering from disorder, defects, etc. As shown in Supplementary Figure S4, the elastic signal is nearly absent and constant with temperature down to 170 K. Below 150 K, a quasi-elastic central peak emerges in the fluctuation regime due to nucleation of nano-domains of the low temperature phase.

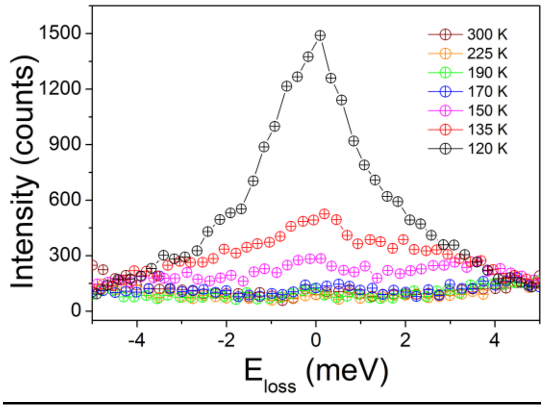

Supplementary Fig. 4: **Temperature dependence of the central peak.** E_{loss} scans at selected temperatures of the elastic central peak. The elastic line is nearly absent upon cooling down to 170 K, indicating low disorder.

2.2) Analysis of the phonon spectra.

In the following, we show the representative fitting of the IXS spectra at 300 and 150 K using damped harmonic oscillators (DHO) for phonons convoluted with the experimental resolution. The dynamic structure factor $S(\mathbf{Q},\omega)$ is given by [1]:

$$S(Q,\omega) = \frac{[n(\omega)+1]Z(Q)4\omega^{\Gamma_q}/_{\pi}}{[(\omega-\omega_q)^2+\Gamma_q^2][(\omega+\omega_q)^2+\Gamma_q^2]} \quad \text{Supplementary Eq. (2)}$$

where $Z(Q)=\exp(-2W_Q)|\boldsymbol{Q}.e|^2/2M$, with the exponential being the Debye-Waller factor, e is the polarization vector and M is the mass of the atom.

In order to understand how many phonon peaks are expected in IXS along the $\boldsymbol{Q} = (2 + h\ 0\ 0.7)$ wavevector, we calculate the

$$F_\mu(\boldsymbol{Q}) = \left|\sum_s \boldsymbol{Q}.\frac{\varepsilon_{\mu s}(\boldsymbol{q})}{\sqrt{M_s}}\right|^2 \quad \text{Supplementary Eq. (3)}$$

factor, which is proportional to the structure factor observed experimentally associated to a phonon mode μ. In the equation above, $\boldsymbol{q}$ is the vector that occurs when $\boldsymbol{Q}$ is brought to the first Brillouin zone, $\boldsymbol{\varepsilon}_{\mu s}(\boldsymbol{q})$ is the polarization vector of mode μ for atom s with mass M_s. For the results presented in Supplementary Figure S5, the calculated harmonic polarization vectors are used. As it can be seen, the mode ω_3 does not provide any intensity along $\boldsymbol{Q} = (2 + h\ 0\ 0.7)$. The other two acoustic modes, ω_1 and ω_2, are visible in principle, though the intensity of the mode ω_2 is expected to be very weak at $h = 0.05$.

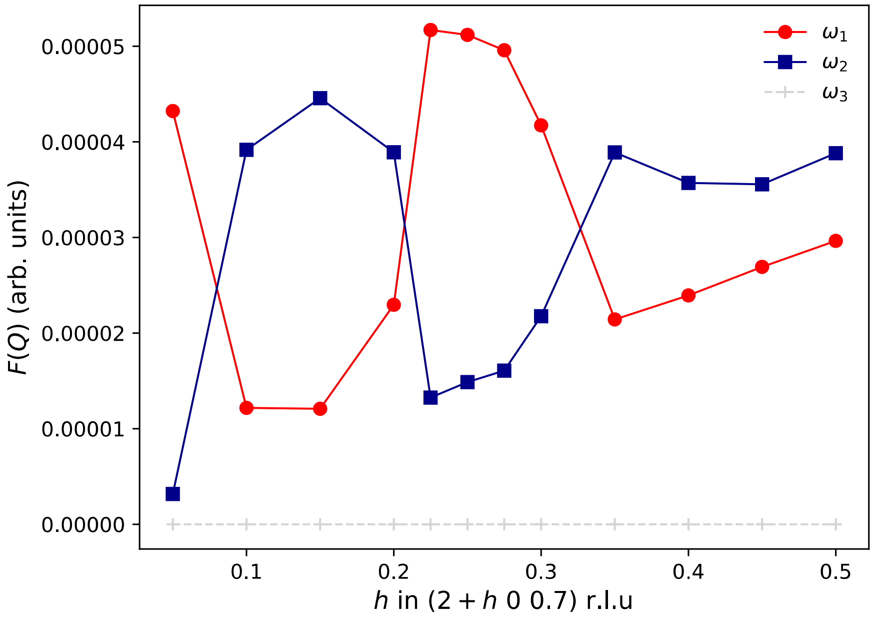

Supplementary Fig. 5: **Structural factor, $F_\mu(Q)$.** Theoretical calculation of the structural factor for the three acoustic modes along $\boldsymbol{Q} = (2 + h\ 0\ 0.7)$.

2.2.1) <u>300 K</u>

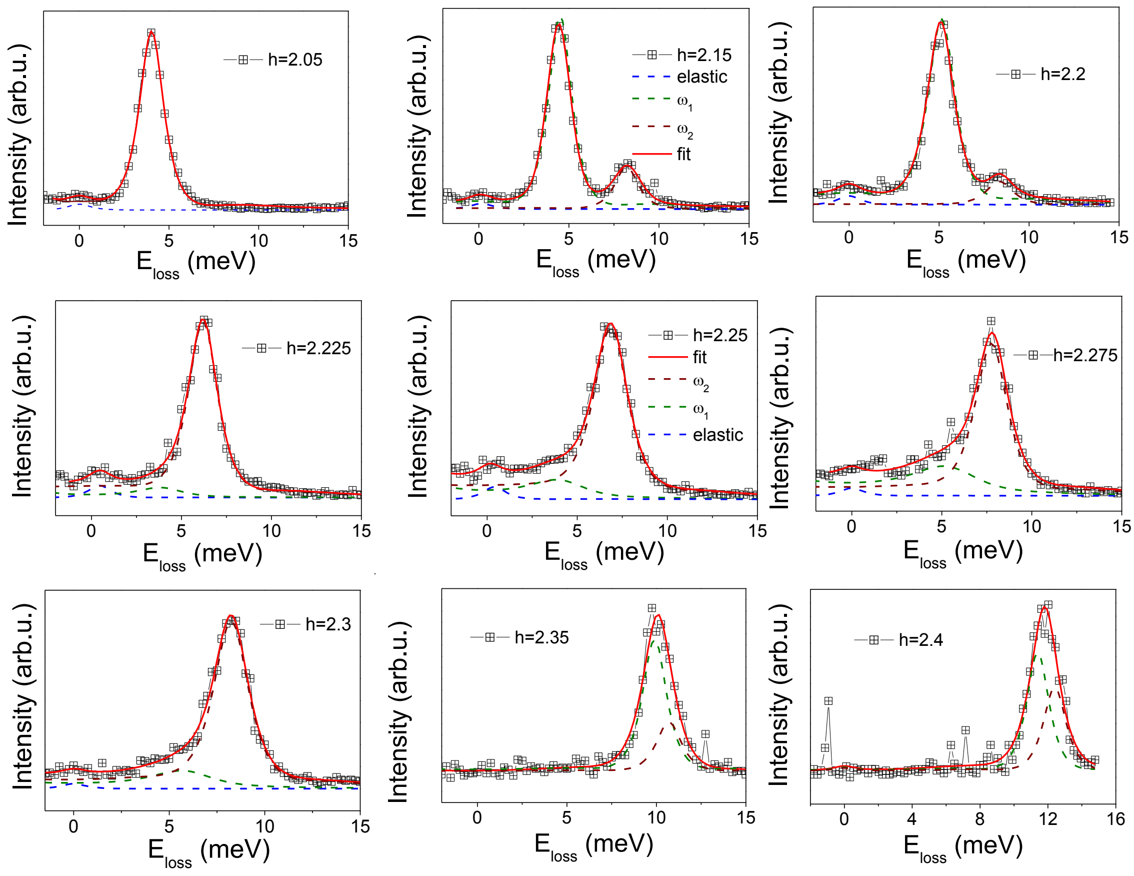

Supplementary Fig. 6: **IXS spectra at 300 K**. Detailed fitting of the experimental IXS spectra its corresponding phonon assigment to ω_1 and ω_2. Each panel corresponds to a different wave number $\boldsymbol{Q} = (h\ 0\ 0.7)$. The dispersion and linewith (full-width at half-maximun) are displayed in the main manuscript.

2.2.2) <u>225 K</u>

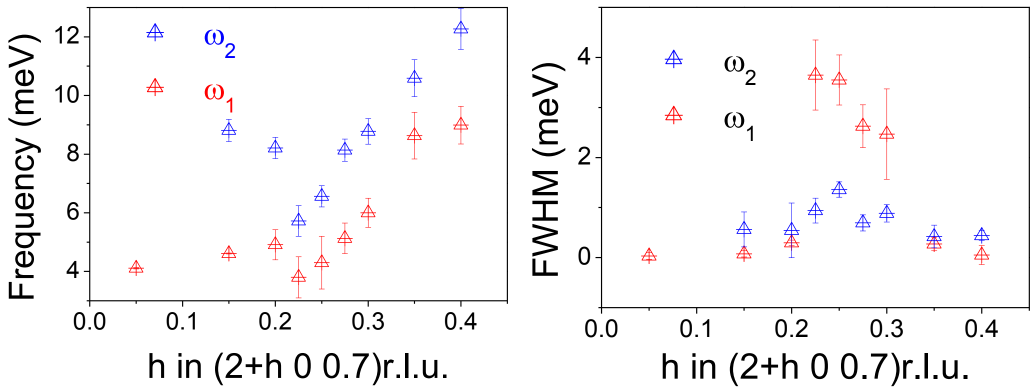

Supplementary Fig. 7: **IXS spectra at 225 K**. Phonon dispersion (left panel) and linewidth (right panel) at 225K. Blue and red tirangles in the left panel represent the frequency of ω_1 and ω_2 low energy phonons. In the right panel, blue and red triangles stand for the full width at half maximum of ω_1 and ω_2. The error bars in the experimental data points in both panels represent the fit uncertainty.

2.2.3) <u>150 K</u>

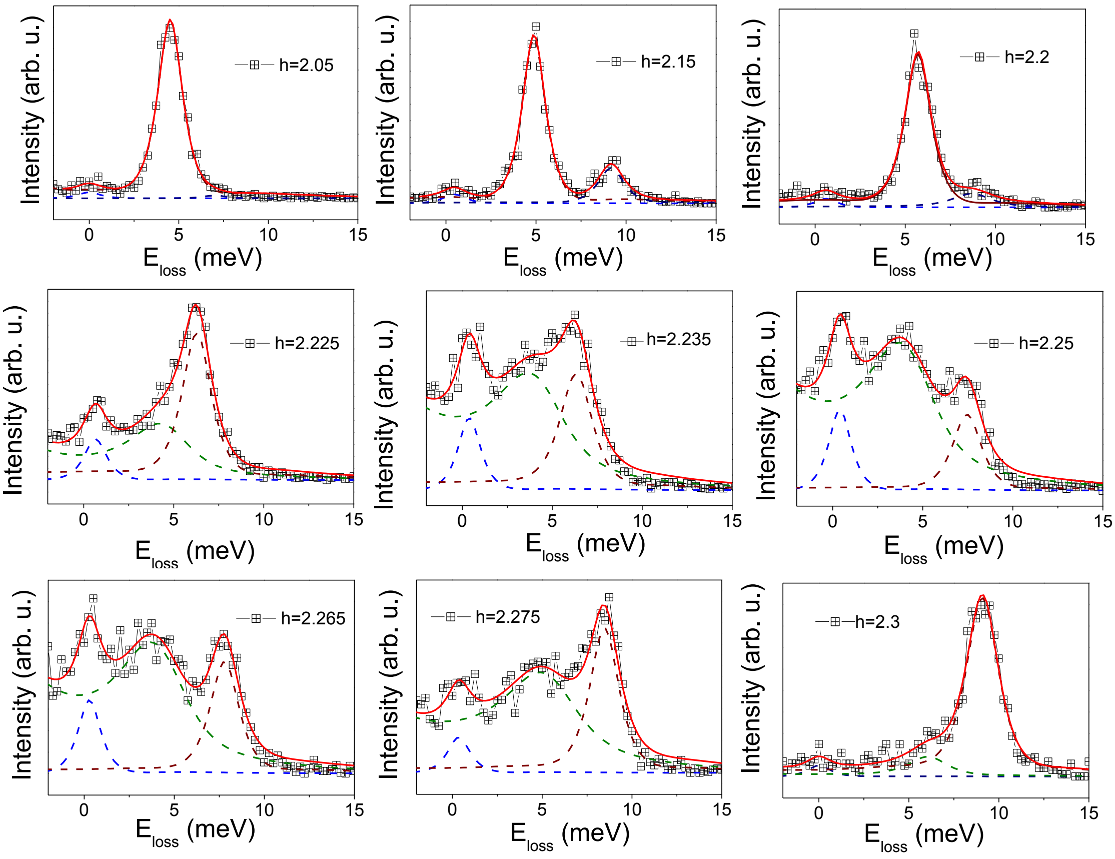

Supplementary Fig. 8: **IXS spectra at 150 K**. Detailed fitting of the experimental IXS spectra at 150 K. Each panel corresponds to a different wave number $\boldsymbol{Q} = (h\ 0\ 0.7)$. The dispersion and linewidth (full-width at half-maximun) are displayed in the main manuscript.

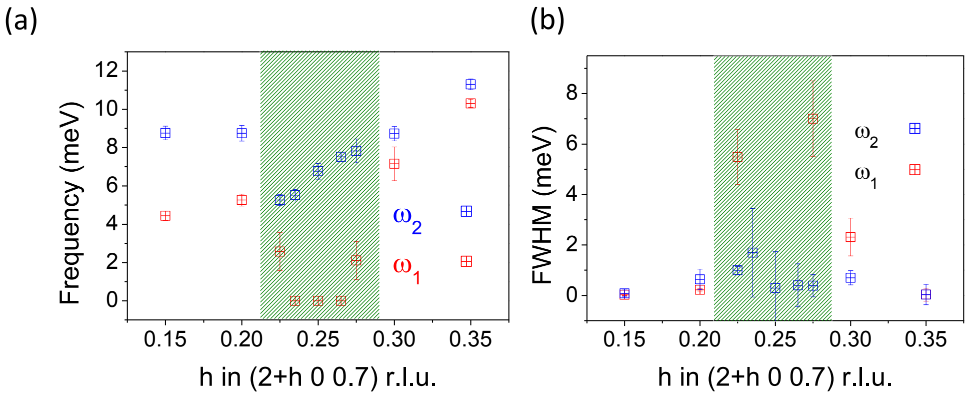

Supplementary Fig. 9: **Frequency and linewidth of the acoustic branch at T_{CDW}=110 K.** Experimental dispersion (a) and linewidth (b) of the low energy acoustic ω_1 and ω_2 phonons at 110 K. Shaded green area denotes the momentum spread of the phonon anomalies at Q_{CDW}. Blue and red squares in the left panel represent the frequency of ω_1 and ω_2 low energy phonons. In the right panel, blue and red squares stand for the full width at half maximum of ω_1 and ω_2. The error bars in the experimental data points in both panels represent the fit uncertainty.

3) *Ab initio* calculation details

- Harmonic and anharmonic calculations:

The anharmonic temperature-dependent phonon frequencies were calculated within the Stochastic Self-Consistent Harmonic Approximation (SSCHA) [2-4]. The SSCHA is a quantum variational method that minimizes the free energy of the system F with respect to centroid positions R and effective force constants Φ. The SSCHA minimization requires the calculation of forces in supercells. The forces were calculated in 4x4x3 supercells within Density Functional Theory (DFT) making use of the Perdew Burke Ernzerhoff (PBE) [5] parametrization of the exchange-correlation functional. An ultrasoft pseudopotential with 5 electrons in the valence was used for V and a norm-conserving pseudopotential for Se with 6 electrons in the valence. There is no problem combining different types of pseudopotentials as norm-conserving pseudopotentials can be understood as an ultrasoft pseudopotential in which the norm is conserved. In our case convergence was reached with a 40 Ry cutoff for the plane-wave basis and 450 Ry for the density. The Brilluoin zone integrals for the force calculations in the supercell were performed with a 3x3x3 k-point grid (equivalent to a 12x12x9 grid in the unit cell) with a Methfessel-Paxton smearing of 0.01 Ry. It was checked that the SSCHA minimization result was well converged with this k-point grid. Harmonic phonon calculations were performed within Density Functional Perturbation Theory (DFPT), with the same parameters as the force calculations but with a 24x24x16 grid (in the unit cell) for the Brillouin zone integrals. The force calculations needed for the SSCHA, the DFPT harmonic phonon calculations, and the calculation of the electron-phonon matrix elements were performed with the Quantum Espresso [6,7].

The SSCHA calculation in a 4x4x3 grid yields anharmonic dynamical matrices in a commensurate 4x4x3 grid of q points, which includes $q_{CDW} = (0.25\ 0\ -1/3)$ r. l. u. In order to obtain other anharmonic phonon frequencies at other q points along $(h\ 0\ -1/3)$ r. l. u., the following steps were followed. The difference between the anharmonic and the harmonic dynamical matrices was obtained at these 4x4x3 grid. This difference was interpolated to other

q points along $(h\,0\;-1/3)$. Adding the harmonic dynamical matrix calculated explicitly at these points to the interpolated difference, the anharmonic dynamical matrices were estimated at other points not commensurate with the 4x4x3 grid.

The theoretical anharmonic phonon spectra shown in Figs. 1, 2, and 3 of the main text were calculated in the static limit of the SSCHA theory [3], in which the anharmonic dynamical matrices are determined by the Hessian of the SSCHA free energy F. The static limit of the theory is well justified for low-energy acoustic modes. The dynamic extension of the theory in the Lorentzian approximation [3] was used to calculate the anharmonic contribution to the phonon linewidth. The calculation was performed considering phonon-phonon scattering on a 160x160x120 grid. The phonon frequencies and third-order force constants at these points were obtained by Fourier interpolation. A 0.1 cm^{-1} Gaussian smearing was used for the Dirac deltas. Supplementary Figure S10 shows the results obtained along the $(h\,0\;-1/3)$ path for the acoustic modes. Interestingly, the anharmonic linewidth is also largely q dependent and, for the ω_1 mode, it peaks at q_{CDW} as the electron-phonon contribution (see main text). All SSCHA calculations were performed in the so-called bubble approximation (see Ref. [2] for further details of the theory).

- Electron-phonon calculations and susceptibility calculations:

The electron-phonon contribution to the phonon linewidth for mode μ with momentum $\boldsymbol{q}$ was calculated as

$$FWHM_{elph,\mu}(\boldsymbol{q}) = \frac{4\pi\omega_\mu(\boldsymbol{q})}{N_k}\sum_{\boldsymbol{k}nm}\left|g^{\mu}_{n\boldsymbol{k},m\boldsymbol{k}+\boldsymbol{q}}\right|^2 \delta(\epsilon_{n\boldsymbol{k}})\delta(\epsilon_{m\boldsymbol{k}+\boldsymbol{q}}),$$ Supplementary Eq. (4)

where $\omega_\mu(\boldsymbol{q})$ is the frequency of the mode, $\epsilon_{n\boldsymbol{k}}$ the band energy of state $n\boldsymbol{k}$ measured from the Fermi energy, N_k the number of $\boldsymbol{k}$ points in the sum, and $g^{\mu}_{n\boldsymbol{k},m\boldsymbol{k}+\boldsymbol{q}}$ the electron-phonon matrix elements. The latter are calculated within DFPT as

$$g^{\mu}_{n\boldsymbol{k},m\boldsymbol{k}+\boldsymbol{q}} = \sum_{s\alpha}\frac{1}{\sqrt{2M_s\omega_\mu(\boldsymbol{q})}}\varepsilon^{\alpha}_{\mu s}(\boldsymbol{q})\,\langle n\boldsymbol{k}\left|\left[\frac{\partial V_{KS}}{\partial u^{\alpha}_s(\boldsymbol{q})}\right]_0\right|m\boldsymbol{k}+\boldsymbol{q}\rangle,$$ Supplementary Eq. (5)

where $\langle n\boldsymbol{k}|[\partial V_{KS}/\partial u^{\alpha}_s(\boldsymbol{q})]_0|m\boldsymbol{k}+\boldsymbol{q}\rangle$ are the matrix elements of the derivative of the Kohn-Sham potential with respect to the Fourier transformed atomic displacements calculated at equilibrium between the electronic states $n\boldsymbol{k}$ and $m\boldsymbol{k}+\boldsymbol{q}$, and α denotes a Cartesian direction. Note that $FWHM_{elph,\mu}(\boldsymbol{q})$ does not depend on the phonon frequencies. $FWHM_{elph,\mu}(\boldsymbol{q})$ was calculated using a 48x48x32 $\boldsymbol{k}$-point grid and a Gaussian smearing of 0.003Ry for the Dirac deltas.

In order to understand the role of the electron-phonon interaction in the CDW formation we also calculated the real part of the non-interacting susceptibility without considering the matrix elements as

$$\chi_0(\boldsymbol{q}) = P\frac{1}{N_k}\sum_{\boldsymbol{k}nm}\frac{f_{n\boldsymbol{k}}-f_{m\boldsymbol{k}+\boldsymbol{q}}}{\epsilon_{n\boldsymbol{k}}-\epsilon_{m\boldsymbol{k}+\boldsymbol{q}}},$$ Supplementary Eq. (6)

where P denotes the principal value and $f_{n\boldsymbol{k}}$ is the Fermi function of state $n\boldsymbol{k}$. The effect of $\chi_0(\boldsymbol{q})$ is somewhat present in the harmonic phonon frequencies through the real part of the electron-phonon self-energy in the static limit:

$$\Pi_\mu(\boldsymbol{q}) = \frac{1}{N_k}\sum_{\boldsymbol{k}nm}\frac{f_{n\boldsymbol{k}}-f_{m\boldsymbol{k}+\boldsymbol{q}}}{\epsilon_{n\boldsymbol{k}}-\epsilon_{m\boldsymbol{k}+\boldsymbol{q}}}\left|g^{\mu}_{n\boldsymbol{k},m\boldsymbol{k}+\boldsymbol{q}}\right|^2 \text{ Supplementary Eq. (7)}$$

However, the electron-phonon matrix elements have a large importance in $\Pi_\mu(\boldsymbol{q})$. Neglecting the electron-phonon matrix elements, the electron-phonon linewidth is affected on the contrary with the so-called nesting function

$$\zeta(\boldsymbol{q}) = \frac{1}{N_k}\sum_{\boldsymbol{k}nm}\delta(\epsilon_{n\boldsymbol{k}})\delta(\epsilon_{m\boldsymbol{k}+\boldsymbol{q}}) \text{ Supplementary Eq. (8)}$$

which is related to the imaginary part of the non-interacting susceptibility. Thus, the ratio between $\mathrm{FWHM}_{\mathrm{elph},\mu}(\mathrm{q})$ and $\zeta(\mathrm{q})$ estimates the role of the electron-phonon matrix elements. If the latter are assumed to be constant, the ratio would also be a constant.

$\chi_0(\mathrm{q})$ and $\zeta(\mathrm{q})$ were calculated by using maximally localized Wannier Functions (MLWF) for entangled bands [8,9] as implemented in the Wannier90 code [10]. We obtained MLWF by using 9 Wannier functions (3 Se p orbitals, and the following d orbitals for V, d_{xy}, $d_{x^2-y^2}$, $d_{z^2-r^2}$) and a 6x6x4 k-point grid for the k-point integration. $\chi_0(\mathrm{q})$ and $\zeta(\mathrm{q})$ where then calculated with 96x96x32 grids and, for the nesting function, a smearing of 0.04 Ry. In Supplementary Figure S11 we show these two functions along at different $\boldsymbol{q}$ directions in the Brillouin zone. As it can be seen, $\chi_0(\boldsymbol{q})$ not only softens at q_{CDW}, it also shows other softening points not related to the CDW.

- Fermi surface and band structure:

In Supplementary Figure S12 we show that the band calculation with our DFT parameters reproduces accurately the ARPES measurements in Ref. [11]. The dog-bone shape of the Fermi surface can be seen in the calculated Fermi surface as well (see Fig. S13).

(a)

(b)

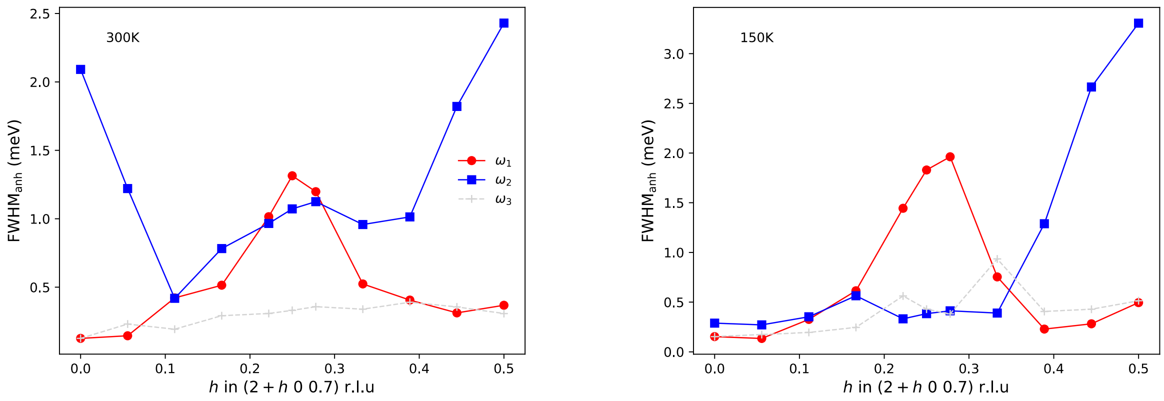

Supplementary Fig. 10: **Anharmonic contribution to the phonon linewidth.** Full width at half maximum (FWHM) at 300 K (a) and at 150 K (b) for the ω_1, ω_2, and ω_3 acoustic modes. Red circles, blue squares and grey crosses stand for ω_1, ω_2 and ω_3, repectively.

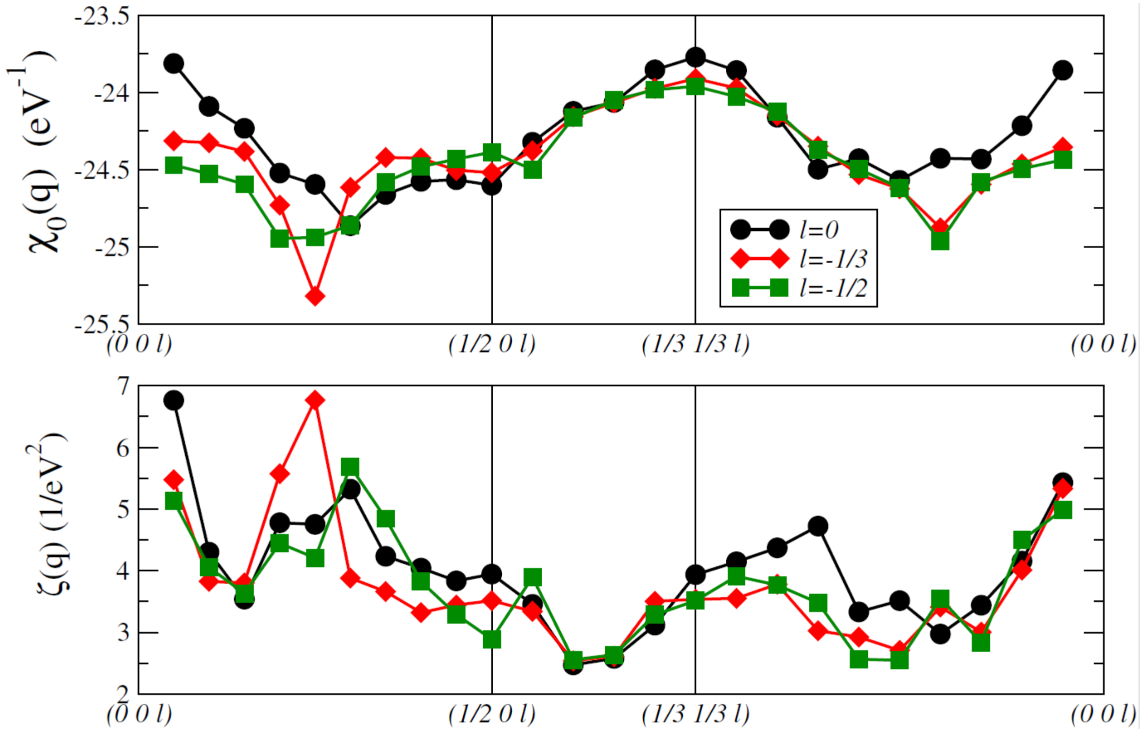

Supplementary Fig.11: **Electronic susceptibility and nesting function**. $\chi_0(\boldsymbol{q})$ (top panel) and $\zeta(\boldsymbol{q})$ (bottom panel) along different $\boldsymbol{q}$ directions of the Brillouin zone. The points are given in r. l. u. For $l = 0$ the path corresponds to $\Gamma MK\Gamma$ and for $l = -1/2$ to ALHA.

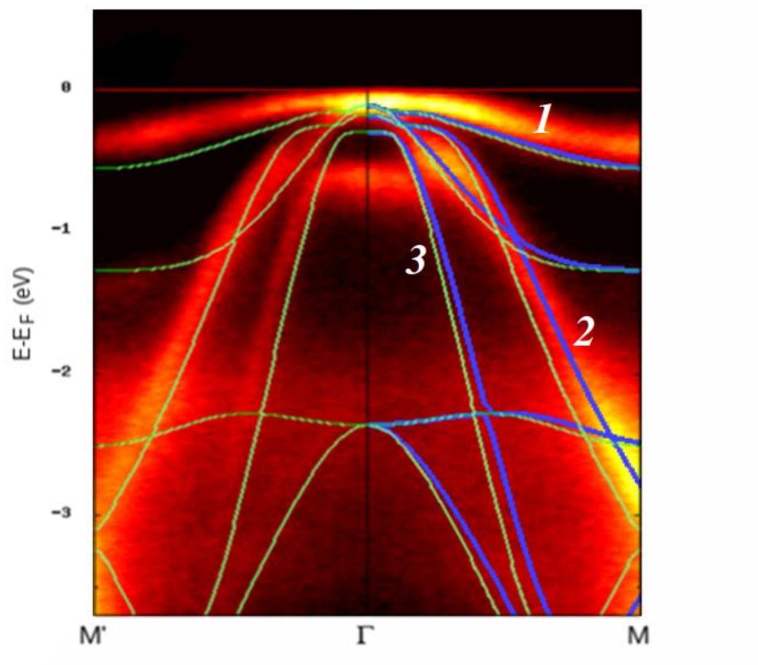

Supplementary Fig. 12: **DFT band structure calculations**. Experimental band structure of VSe_2 along the M'-Γ-M direction and DFT band calculations (blue lines), taken from Ref. [11]. Black to red to yellow indicate a low to high photoemission intensity. Our *ab initio* DFT band structure (green lines) is superimposed to the ARPES measurements of Ref [11].

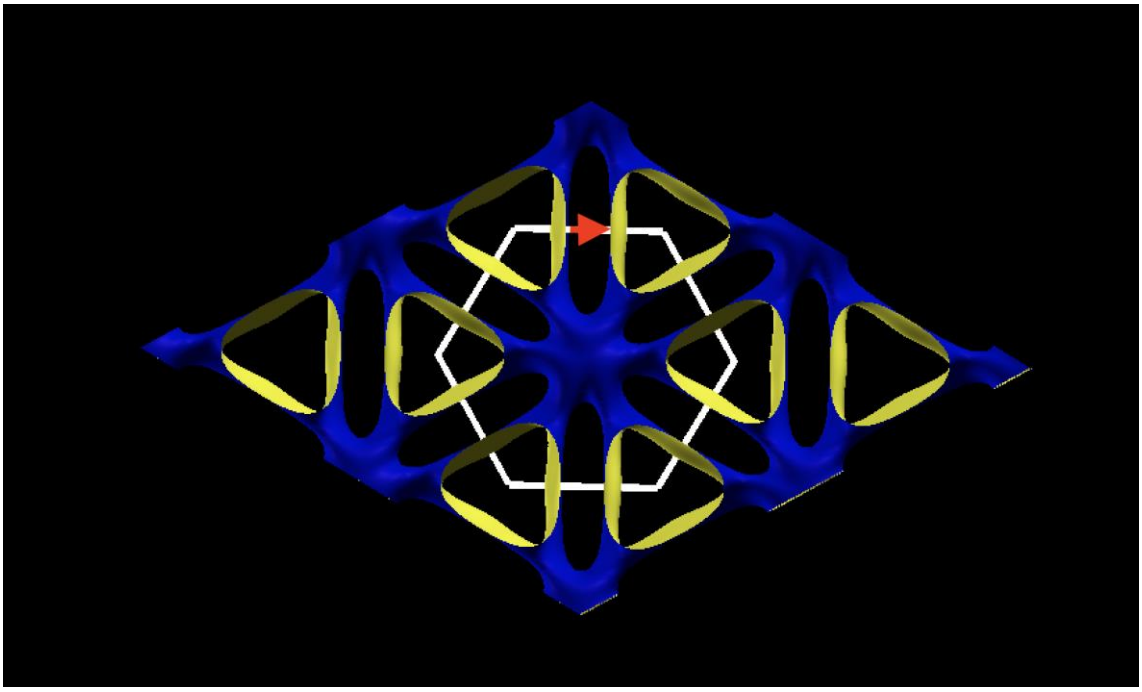

Fig. S13: **Calculated Fermi surface of VSe_2.**

References:

[1] B. Fak, B. Dorner, Physica B 234-236 (1997) 1107-1108

[2] I. Errea, M. Calandra, and F. Mauri, Phys. Rev. B 89, 064302 (2014).

[3] R. Bianco, I. Errea, L. Paulatto, M. Calandra, andF. Mauri, Phys. Rev. B96, 014111 (2017).

[4] L. Monacelli, I. Errea, M. Calandra, and F. Mauri, Phys.Rev. B98, 024106 (2018).

[5] J. P. Perdew, K. Burke, and M. Ernzerhof, Phys. Rev. Lett. 77, 3865 (1996).

[6] P. Giannozzi et al., J. Phys. Condens. Matter 21, 395502 (2009).

[7] P. Giannozzi, O. Andreussi, T. Brumme, O. Bunau, M. B.Nardelli, M. Calandra, R. Car, C. Cavazzoni, D. Ceresoli,M. Cococcioni, N. Colonna, I. Carnimeo, A. D. Corso, S. de Gironcoli, P. Delugas, R. A. D. Jr, A. Ferretti,A. Floris, G. Fratesi, G. Fugallo, R. Gebauer, U. Gerst-mann, F. Giustino, T. Gorni, J. Jia, M. Kawamura, H.-Y.Ko, A. Kokalj, E. Küçükbenli, M. Lazzeri, M. Marsili, N. Marzari, F. Mauri, N. L. Nguyen, H.-V. Nguyen, A. O.de la Roza, L. Paulatto, S. Poncé, D. Rocca, R. Sabatini, B. Santra, M. Schlipf, A. P. Seitsonen, A. Smogunov, I. Timrov, T. Thonhauser, P. Umari, N. Vast, X. Wu, and S. Baroni, Journal of Physics: Condensed Matter29,465901 (2017).

[8] N. Marzari, D. Vanderbilt, Phys. Rev. B 56, 12847 (1997).

[9] I. Souza, N. Marzari, D. Vanderbilt, Phys. Rev. B 65, 035109 (2001).

[10] A. A. Mostofi, J. R. Yates, Y.-S. Lee, I. Souza, D. Vanderbilt, N. Marzari, Computer Physics Communications 178, 685 (2008).

[11] Vladimir N. Strocov, Ming Shi, Masaki Kobayashi, Claude Monney, Xiaoqiang Wang, Juraj Krempasky, Thorsten Schmitt, Luc Patthey, Helmuth Berger, and Peter Blaha, Phys. Rev. Lett. 109, 086401 (2012).
